# The relationship between resting heart rate and new‐onset microalbuminuria in people with type 2 diabetes: An 8‐year follow‐up study

**DOI:** 10.1111/dme.14436

**Published:** 2020-11-18

**Authors:** Y. K. Chang, H. C. Fan, P. S. Lim, S. Y. Chuang, C. C. Hsu

**Affiliations:** ^1^ Department of Medical Research Tungs Taichung MetroHarbor Hospital Taichung Taiwan; ^2^ Department of Nursing Jenteh Junior College of Medicine, Nursing and Management Miaoli Taiwan; ^3^ Department of Pediatrics Tungs Taichung MetroHarbor Hospital Taichung Taiwan; ^4^ Department of Rehabilitation Jenteh Junior College of Medicine, Nursing and Management Miaoli Taiwan; ^5^ Division of Renal Medicine Tungs Taichung MetroHarbor Hospital Taichung Taiwan; ^6^ Institute of Population Health Sciences National Health Research Institutes Miaoli Taiwan; ^7^ Department of Health Services Administration China Medical University Taichung Taiwan; ^8^ Department of Family Medicine Min‐Sheng General Hospital Taoyuan Taiwan

**Keywords:** microalbuminuria, prognostic risk factor, resting heart rate, type 2 diabetes

## Abstract

**Aims:**

Microalbuminuria is an indicator of adverse cardiovascular events and chronic kidney disease. Studies have described an elevated resting heart rate as a risk factor for microalbuminuria in people with cardiovascular disease, but none have clarified its role in microalbuminuria development in people with type 2 diabetes. Therefore, this study investigated the relationship between resting heart rate and new‐onset microalbuminuria in type 2 diabetes.

**Methods:**

A total of 788 people from a glycaemic control trial in Taiwan were enrolled. Microalbuminuria was defined as a fasting urine albumin‐to‐creatinine ratio ≥30 mg/g in two consecutive urine tests. Resting heart rate and other covariates were measured at baseline. The quartile of resting heart rates, categorized as <70, 70–74, 75–80 and >80 beats/min, was used for analysis. Cox proportional hazard models were used to evaluate the association between resting heart rate and risk of microalbuminuria.

**Results:**

During the follow‐up period, 244 people (31%) developed microalbuminuria. Those who developed microalbuminuria had a longer diabetes duration (median = 3.0 vs. 2.0 years, *p* < 0.001), higher rate of hypertension (77% vs. 66%, *p* = 0.003), higher rate of angiotensin‐converting enzyme inhibitor/angiotensin receptor blocker treatment (50% vs. 38%, *p* = 0.001) and higher baseline HbA_1c_ level (70 vs. 64 mmol/mol, 8.6 vs. 8.0%, *p* < 0.001). After adjusting for demographics, metabolic profiles and inflammatory markers, developing microalbuminuria was significantly associated with baseline resting heart rate of 70–74, 75–80 and >80 beats/min (with hazard ratios [95% CI] of 2.05 [1.32, 3.18], 2.10 [1.32, 3.32] and 1.62 [1.01, 2.59], respectively) compared to resting heart rates <70 beats/min. An average increased risk of microalbuminuria for increment of 10 beats/min was about 24% among those with hypertension (with hazard ratios of 1.24 [1.05, 1.47] in the multivariable Cox model).

**Conclusions:**

This prospective cohort study showed that resting heart rate may be an associative risk factor for developing microalbuminuria in type 2 diabetes.

**Novelty statement**

- An increased resting heart rate has been described as a risk predictor of microalbuminuria among people with cardiovascular disease, but there is limited research on the link in people with type 2 diabetes.
- This study revealed that, particularly among those with hypertension, the risk of microalbuminuria increases with increment of heart beat.
- This prospective cohort study showed that resting heart rate can be an independent prognostic risk factor for the future development of microalbuminuria in people with type 2 diabetes.

## 1 INTRODUCTION

In addition to being a widely recognized indicator of autonomic nervous system tone, resting heart rate has further been found to have significant correlations with metabolic disturbances, increased body mass index and high blood pressure, with these associations being particularly notable in people with diabetes or hypertension.^1^ It has also been found to be associated with mortality in a wide range of populations, including large clinical and epidemiological populations of healthy individuals, as well as people suffering from coronary artery disease, hypertension, left ventricular systolic dysfunction and chronic heart failure.^2^, ^3^, ^4^ In addition, associations between increased resting heart rate and cardiovascular events in people with type 2 diabetes, such as cardiovascular death, stroke, heart failure and myocardial infarction, have also been demonstrated in previous research.^5^ The association between resting heart rate and mortality appears to be independent of traditional cardiac risk factors and could be of value in clarifying how individual risk is not sufficiently accounted for in models not taking it into account.

One of the organs most strongly targeted by metabolic diseases is the kidney. Chronic kidney disease (CKD), a condition that mainly results from diabetes and hypertension,^6^ can be exacerbated or recur due to ill‐timed diagnosis and treatment and can lead, in turn, to deteriorated renal function, anaemia, abnormal bone metabolism, greater risk of cardiovascular disease, and cognitive disorders. The levels of microalbumin in urine constitute one sensitive biomarker of early renal function injury,^7^ while past studies have also demonstrated that albuminuria is an independent risk factor not only for cerebral and cardiovascular diseases but also stroke and cardiocerebrovascular mortality.^8^, ^9^ Furthermore, microalbuminuria is a biomarker of heightened vascular permeability and is correlated with an increased probability of cardio‐renal‐vascular disease and death.^10^ The presence of microalbuminuria, meanwhile, is relatively common in people with type 2 diabetes, reportedly developing in approximately 2% per year after initial type 2 diabetes diagnosis.^11^

In hypertensive people with elevated cardiovascular risk, one independent predictor for the development of microalbuminuria is an elevated heart rate.^12^ Past studies have demonstrated, relatedly, that hypertension treatments can be effective in reducing the occurrence of microalbuminuria, with the most effective of these treatments being renin–angiotensin system blockers.^13^ Research has further demonstrated that the use of pharmaceuticals to lower heart rates in mice caused improvements in their endothelial function,^14^ suggesting that this approach could also potentially be applied to reduce the development of microalbuminuria in humans. At the same time, the means by which the development of microalbuminuria is affected by heart rate in diabetes people is still not clear, though it seems probable that a mixture of factors plays a role in its development, with elevated levels of base membrane permeability, glomerular pressure, inflammatory effects, pulse waves, and proatherosclerotic activity having all been hypothesized to play a part.^12^, ^15^

In the present study, we analysed data from an earlier 10‐year follow‐up study of people with type 2 diabetes in order to clarify the relationship between resting heart rate and new‐onset microalbuminuria.

## 2 METHODS

### 2.1 Study design and participants

The data utilized in this study were sourced from people with type 2 diabetes who took part in the diabetes management through an integrated delivery system (DMIDS) project (ClinicalTrials.gov, NCT00288678), which was the first randomized clinical trial in Taiwan focusing on evaluating the effectiveness of an integrated care programme for people with diabetes. After the initial 5‐year intervention study, which lasted from 2003 to 2007, the DMIDS project was transformed into an observational cohort study (for the period from 2008 to 2012) in order to observe the risk factors related to diabetic nephropathy. Any individual diagnosed with type 2 diabetes according to the ADA guideline was eligible to participate unless they met any of the following exclusion criteria: (1) being younger than 30 or older than 70 years of age; (2) being pregnant; or (3) having major diabetic complications, such as uraemia, a leg amputation or hospitalization within the preceding year due to heart failure, acute myocardial infarction, or stroke.^16^ A total of 36 local clinics in northern, central and southern Taiwan were chosen as the study sites, and the participants were followed up until 31 December 2012. The Institutional Review Board of the National Health Insurance Institutes (EC 0970302) reviewed and approved the present study.

### 2.2 Laboratory tests

Fasting (overnight for at least 8 h) venous blood and spot urine specimens were collected every 6 months. High performance liquid chromatography (Variant II; Bio‐Rad Laboratories) was used to measure glycated haemoglobin (HbA_1c_) levels, while an automatic analyser (Hitachi 7060; Hitachi High Technologies) was used to measure fasting glucose, triglyceride, LDL and HDL cholesterol levels. Insulin levels were measured using a chemiluminescent immunometric assay (Immulite1000; Diagnostic Products), urinary albumin levels were measured using the immunoturbidimetric method (Hitachi7060; Hitachi High Technologies), and dipstick urinalysis was performed with an automated chemical analyser (Clinitek 500; Bayer). All blood and urine samples were kept in temperature‐controlled containers at 2°C to 8°C, transported by express delivery to a central laboratory and measured within 8 h.

### 2.3 Variable definitions and anthropometric measurements

The baseline data of the participating people, including their demographic variables (gender, age, age at diabetes diagnosis, education level, duration of diabetes, smoking history and drinking status), baseline biochemical indicators (HbA_1c_, cholesterol, triglyceride level, hypertension, systolic blood pressure, diastolic blood pressure, uric acid, HOMA‐IR, high‐sensitivity C‐reactive protein [hsCRP] level, eGFR and electrocardiogram [ECG] abnormal status), and oral anti‐hypertension and oral anti‐diabetes agent use were collected by trained health workers via standardized procedures. The HOMA of insulin resistance (HOMA‐IR) (HOMA‐IR = insulin [mU/L] × fasting glucose [mmol/L]/22.5) was used to assess insulin resistance.^17^ ECG measurements, including any ECG abnormalities and the type of each abnormality, were interpreted by a commissioned cardiologist according to the Minnesota Code classification system,^18^ with the ECG findings for each patient being classified as major, minor or no abnormalities. The ECG classified as major or minor abnormalities was considered an abnormal ECG. The primary endpoint of this study was the development of microalbuminuria. The blood pressure and resting heart rate of each participant were measured three times in the participant's non‐dominant arm while he or she was seated using an automated electronic device (OMRON Model HEM‐725 FUZZY, Omron Company). The three measurements were made consecutively at 1‐min intervals after a rest of ≥5 min, with the three measurements then averaged for subsequent analysis. The participants were stratified by the quartiles of the resting heart rate into four groups (< 70, 70–74, 75–80 and >80 beats/min). The participants who had a fasting urine albumin‐to‐creatinine ratio (ACR) of at least 30 mg/g in two consecutive urine tests were regarded as having developed microalbuminuria. However, a given urine sample was excluded from the analysis if a microscopic urinalysis showed erythrocytes >5/high‐power field (HPF), white blood cells >5/HPF, epithelial cells >5/HPF and the presence of casts or bacteria. A participant who had smoked <100 cigarettes in their life was defined as a non‐smoker, while a participant who had smoked ≥100 cigarettes was defined as an ex‐smoker if he or she had stopped smoking completely ≥1 month before being recruited; and was defined as a current smoker if he or she had a daily or occasional smoking habit when recruited.

### 2.4 Statistical analysis

The dependent variable was time to development of microalbuminuria. The person‐years were calculated as the total time passed from the date of recruitment until the date of death, the development of microalbuminuria, or the final follow‐up, whichever came first. Differences in characteristics between the people with and without microalbuminuria were determined using the Student's *t‐*test (for means of continuous variables), Mann–Whitney *U‐*test (for medians of continuous variables) or the chi‐squared test (for categorical variables). The baseline resting heart rate values were used to divide the total population of people into quartiles, with the lowest quartile used as the reference and the ANOVA test and Kruskal–Wallis test were used for comparisons of means and medians respectively. Hazard ratios and corresponding 95% confidence intervals (CIs) were calculated using Cox proportional hazard regression. It was confirmed, through utilization of the Schoenfeld residuals test^19^ and complementary log–log plots, that the proportional hazards assumption for all covariates was not violated. Both unadjusted and adjusted data are presented for the covariates of gender, age, age at diabetes diagnosis, education level, duration of diabetes, smoking history, drinking status, BMI, waist circumference, HbA_1c_, LDL cholesterol, triglyceride level, hypertension, systolic blood pressure, diastolic blood pressure, uric acid, HOMA‐IR, oral anti‐diabetes agent use, oral anti‐hypertension agent use, statin use, hsCRP level, CKD (eGFR<60 ml/min/1.73 m^2^) and ECG abnormal status. The data were analysed using SAS version 9.4 (SAS Institute Inc.).

## 3 RESULTS

**Figure 1:**
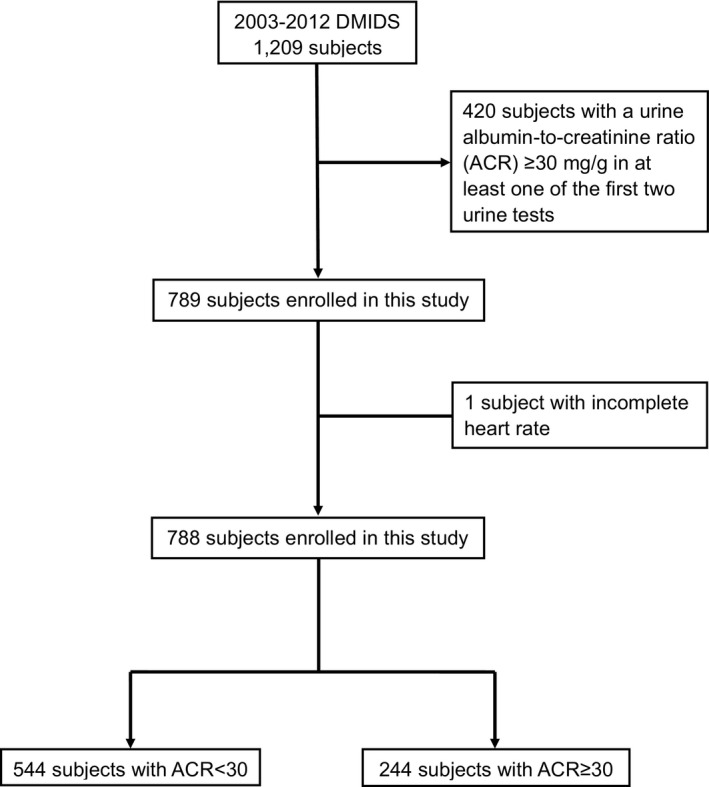
The flow chart of this study

A total of 788 people from the DMIDS study were included after excluding 420 participants with a urine ACR ≥30 mg/g in at least one of the first two urine tests and one person with incomplete heart rate data. Of those 788 people, there were 244 participants who had new‐onset microalbuminuria and 544 participants who did not have microalbuminuria within the median follow‐up duration of 7.9 years. The flow chart of this study is shown in Figure 1. Comparisons of the baseline characteristics for the people with and without microalbuminuria development are shown in Table 1. Overall, the people had a mean age of 55.6 ± 8.6 years, and 47% were male. The mean age at diabetes diagnosis and the median (Q1–Q3) duration of diabetes were 51.1 ± 8.9 and 3.0 (1.0–6.0) years respectively. The mean BMI was 25.8 ± 3.6 kg/m^2^, and 70.1% were non‐smokers. The mean waist circumference was 87.2 ± 10.1 cm. In terms of blood pressure, the mean systolic blood pressure was 128.0 ± 15.8 mmHg, and the mean diastolic blood pressure was 81.7 ± 7.1 mmHg. The mean of uric acid and median (Q1–Q3) of HOMA‐IR were 5.8 ± 1.7 mg/dl (347 ± 101 µmol/L) and 2.8 (1.3–4.8) respectively. The mean of HbA_1c_ and median (Q1–Q3) of hsCRP were 66 ± 20 mmol/mol (8.2 ± 1.8%) and 0.17 (0.08–0.38) mg/dl (1.6 [0.8–3.6] nmol/L) respectively. The percentages of those with eGFR<60 ml/min/1.73 m^2^ and ECG abnormal status were 4.8% and 16% respectively. Compared to people without microalbuminuria, people with microalbuminuria had a longer mean duration of diabetes, a higher mean HbA_1c_ level, a higher rate of hypertension, a higher mean systolic blood pressure, a higher mean HOMA‐IR level and a higher rate of angiotensin‐converting enzyme inhibitor/angiotensin II receptor blocker (ARB) use.

**Table 1:** Comparison of baseline characteristics of patients with and without microalbuminuria (MAU)

	Overall (*n* = 788)	Without MAU (*n* = 544)	With MAU (*n* = 244)	*p* value
Male, *n* (%)	366 (47)	253 (47)	113 (47)	0.959
Age (mean ± SD)	55.6 ± 8.6	55.6 ± 8.4	55.5 ± 8.8	0.882
Age at diabetes diagnosis (mean ± SD)	51.1 ± 8.9	51.6 ± 8.7	50.1 ± 9.2	0.033
Education ≤6 years, *n* (%)	359 (45.6)	256 (47.1)	103 (42.2)	0.207
Duration of diabetes, years (median, Q1–Q3)	3.0, 1.0–6.0	2.0, 1.0–5.0	3.0, 1.0–8.0	<0.001
Smoking status, *n* (%)				
Non‐smoker	552 (70)	387 (71)	165 (68)	0.319
Ex‐smoker/Current smoker	236 (30.0)	157 (29)	79 (32)	
Drinking status, *n* (%)				
Non‐drinking	515 (65)	349 (64)	166 (68)	0.290
Moderate/Heavy drinker	273 (35)	195 (36)	78 (32)	
BMI, kg/m^2^ (mean ± SD)	25.8 ± 3.6	25.8 ± 3.7	25.7 ± 3.5	0.781
Waist circumference, cm (mean ± SD)	87.2 ± 10.1	87.5 ± 10.5	86.5 ± 9.2	0.215
HbA_1c_, mmol/mol (mean ± SD, %)	66 ± 20 (8.2 ± 1.8)	64 ± 19 (8.0 ± 1.8)	70 ± 21 (8.6 ± 1.9)	<0.001
LDL cholesterol, mg/dl or on drug treatment (mean ± SD, mmol/L)	123.3 ± 33.6 (3.2 ± 0.9)	122.9 ± 33.9 (3.1 ± 0.9)	124.4 ± 32.9 (3.2 ± 0.9)	0.553
Triglycerides, median, Q1–Q3, mg/dl (median, Q1–Q3, mmol/L)	127.0, 91.0–184.0 (1.4, 1.0–2.1)	124.0, 91.0–177.0 (1.4, 1.0–2.0)	133.0, 94.5–188.5 (1.5, 1.1–2.2)	0.100
Hypertension, *n* (%)	547 (69)	360 (66)	187 (77)	0.003
Systolic blood pressure, mmHg (mean ± SD)	128.0 ± 15.8	127.2 ± 15.3	129.7 ± 16.8	0.036
Diastolic blood pressure, mmHg (mean ± SD)	81.7 ± 7.1	82.1 ± 6.9	80.8 ± 9.6	0.599
Uric acid, mg/dl (mean ± SD, µmol/L)	5.8 ± 1.7 (347 ± 101)	5.8 ± 1.7 (346 ± 102)	5.8 ± 1.6 (348 ± 98)	0.873
HOMA‐IR (median, Q1–Q3)	2.8, 1.3–4.8	2.7, 1.3–4.4	3.0, 1.4–5.0	0.219
Oral anti‐diabetes agent use, *n* (%)				
Sulphonylurea	706 (90)	481 (88)	225 (92)	0.107
Biguanide	635 (81)	428 (77)	207 (85)	0.043
Thiazolidinedione	83 (11)	56 (10)	27 (11)	0.744
α‐Glucosidase inhibitor	44 (5.6)	27 (5.0)	17 (7.0)	0.257
Meglitinide	18 (2.3)	11 (2.0)	7 (2.9)	0.462
Oral anti‐hypertension agent use, *n* (%)				
Angiotensin‐converting enzyme inhibitor/angiotensin II receptor blocker	325 (41)	204 (38)	121 (50)	0.001
Calcium channel blocker	261 (33)	174 (32)	74 (37)	0.312
β‐Blocker	252 (32)	167 (31)	85 (35)	0.250
Diuretics	155 (20)	102 (19)	53 (22)	0.332
Statin use, *n* (%)	23 (2.9)	18 (3.3)	5 (2.1)	0.331
hsCRP, median, Q1–Q3, mg/dl (median, Q1–Q3, nmol/L)	0.17, 0.08–0.38 (1.6, 0.8–3.6)	0.16, 0.08–0.38 (1.5, 0.7–3.6)	0.18, 0.09–0.36 (1.7, 0.9–3.4)	0.317
eGFR < 60 ml/min/1.73 m^2^, *n* (%)	38 (4.8)	28 (5.2)	10 (4.1)	0.525
ECG abnormal, *n* (%)	126 (16)	82 (15)	44 (18)	0.295
Heart rate (mean ± SD)	75.8 ± 10.0	75.4 ± 9.9	76.8 ± 10.2	0.075

Abbreviation

MAU, microalbuminuria.

The people were divided in terms of resting heart rate at baseline into groups with <70, 70–74, 75–80 and >80 beats/min. A comparison of baseline characteristics of these four groups is shown in Table 2. There were significant differences between these four groups in terms of LDL cholesterol levels and rates of being on drug treatment, triglyceride levels, hsCRP levels, rates of angiotensin‐converting enzyme inhibitor/ARB use, rates of diuretic use, rates of statin use and rates of ECG abnormal status.

**Table 2:** Comparison of characteristics among patients stratified by heart rate at baseline examination

	<70	70–74	75–80	>80	*p* value
	(*n* = 180)	(*n* = 221)	(*n* = 185)	(*n* = 202)	
Male, *n* (%)	88 (49)	114 (52)	79 (43)	85 (42)	0.146
Age (mean ± SD)	56.7 ± 8.3	55.3 ± 9.2	54.9 ± 8.2	55.5 ± 8.4	0.217
Age at diabetes diagnosis (mean ± SD)	52.8 ± 8.6	50.7 ± 9.4	50.4 ± 8.8	50.9 ± 8.5	0.058
Education ≤6 years, *n* (%)	79 (44)	103 (47)	84 (45)	93 (46)	0.763
Duration of diabetes, years (median, Q1–Q3)	3.0, 1.0–5.0	3.0, 1.0–7.0	3.0, 1.0–6.0	3.0, 1.0–7.0	0.883
Smoking status, *n* (%)					
Non‐smoker	123 (68)	155 (70)	129 (70)	145 (72)	0.504
Ex‐smoker/Current smoker	57 (32)	66 (30)	56 (30)	57 (28)	
Drinking status, *n* (%)					
Non‐drinking	120 (67)	128 (58)	124 (67)	143 (71)	0.124
Moderate/Heavy drinker	60 (33)	93 (42)	61 (33)	59 (29)	
BMI, kg/m^2^	25.7 ± 3.6	26.1 ± 3.7	25.4 ± 3.3	25.8 ± 3.8	0.262
Waist circumference, cm	88.1 ± 10.8	88.5 ± 9.8	85.6 ± 9.2	86.3 ± 10.4	0.011
HbA_1c_, mmol/mol (mean ± SD, %)	64 ± 19 (8.0 ± 1.7)	65 ± 21 (8.1 ± 1.9)	68 ± 20 (8.4 ± 1.8)	67 ± 19 (8.3 ± 1.8)	0.309
LDL cholesterol, mg/dl or on drug treatment (mean ± SD, mmol/L)	120.8 ± 32.5 (3.1 ± 0.8)	127.2 ± 33.9 (3.3 ± 0.9)	126.4 ± 34.7 (3.3 ± 0.9)	118.5 ± 32.4 (3.1 ± 0.8)	0.022
Triglycerides, median, Q1–Q3, mg/dl (median, Q1–Q3, mmol/L)	127.0, 90.5–177.5 (1.4, 1.0–2.0)	124.5, 83.0–179.5 (1.4, 0.9–2.0)	120.0, 90.0–185.0 (1.4, 1.0–2.1)	133.5, 100.0–190.5 (1.5, 1.1–2.2)	0.184
Hypertension, *n* (%)	102 (57)	101 (46)	88 (48)	111 (55)	0.074
Systolic blood pressure, mmHg	128 ± 16.9	126 ± 14.7	128.6 ± 15.5	129.5 ± 16.1	0.124
Diastolic blood pressure, mmHg	79.6 ± 10	79.1 ± 8.4	80.1 ± 9.2	88 ± 100.8	0.266
Uric acid, mg/dl (mean ± SD, µmol/L)	6.1 ± 1.9 (363 ± 113)	5.8 ± 1.8 (345 ± 106)	5.6 ± 1.5 (335 ± 90)	5.8 ± 1.6 (345 ± 92)	0.061
HOMA‐IR (median, Q1–Q3)	2.9, 1.3–4.9	2.8, 1.3–4.8	2.6, 1.4–4.1	2.7, 1.1–4.9	0.810
Oral anti‐diabetes agent use, *n* (%)					
Sulphonylurea	156 (87)	195 (88)	168 (91)	187 (93)	0.224
Biguanide	146 (81)	172 (78)	150 (81)	167 (83)	0.638
Thiazolidinedione	16 (8.9)	25 (11)	17 (9)	25 (12)	0.628
a‐Glucosidase inhibitor	11 (6.1)	11 (5)	13 (7)	9 (4.5)	0.690
Meglitinide	4 (2.2)	5 (2.3)	3 (1.6)	6 (3)	0.851
Oral anti‐hypertension agent use, *n* (%)					
Angiotensin‐converting enzyme inhibitor/angiotensin II receptor blocker	90 (50)	77 (35)	65 (36)	93 (46)	0.003
Calcium channel blocker	72 (40)	72 (33)	54 (29)	63 (31)	0.138
β‐Blocker	67 (37)	70 (32)	48 (26)	67 (33)	0.138
Diuretics	45 (25)	36 (16)	24 (13)	50 (25)	0.004
Statin use, *n* (%)	4 (2.2)	2 (0.9)	5 (2.7)	12 (5.9)	0.019
hsCRP, median, Q1–Q3, mg/dl (median, Q1–Q3, nmol/L)	0.18, 0.09–0.37 (1.7, 0.8–3.5)	0.16, 0.08–0.34 (1.5, 0.8–3.2)	0.14, 0.08–0.30 (1.3, 0.8–2.8)	0.19, 0.09–0.49 (1.8, 0.9–4.7)	0.083
eGFR<60 ml/min/1.73 m^2^, *n* (%)	13 (7.2)	12 (5.4)	5 (2.7)	8 (4.0)	0.206
ECG abnormal, *n* (%)	42 (23)	40 (18)	25 (14)	19 (9.4)	0.002

The Cox proportional hazards regression analysis results for microalbuminuria risk are shown in Table 3. We found that people with an HbA_1c_ level ≥9% had a higher risk of developing microalbuminuria than those with an HbA_1c_ level <7% after adjusting for the related covariates listed in Table 1 (hazard ratio: 1.92, 95% CI:1.26, 2.93). In the same multivariable analysis, the results showed that people with a HOMA‐IR between 3.5 and 4.9 had a higher risk of microalbuminuria than those with a HOMA‐IR <1.5 after adjusting for the related covariates. On the other hand, people with an eGFR level <60 ml/min/1.73 m^2^ had a higher risk of microalbuminuria than those with an eGFR level ≥60 ml/min/1.73 m^2^. In comparison to the people with an heart rate <70 beats/min, the risk of having microalbuminuria was increased among those with heart rates of 70–74, 75–80 and >80 beats/min, with associated hazard ratios of 2.05 (95% CI 1.32, 3.18), 2.10 (95% CI 1.32, 3.32) and 1.62 (95% CI 1.01, 2.59) respectively.

**Table 3:** Univariate and adjusted Cox proportional hazards model results

	Univariate HR	*p*	Multivariable HR	*p*
Male	0.91 (0.68, 1.21)	0.504	1.03 (0.69, 1.56)	0.873
Education (≤6 years)	1.15 (0.87, 1.53)	0.333	1.17 (0.83, 1.63)	0.375
Age at DM onset (per 1 year increment)	0.98 (0.97, 1.00)	0.027	0.99 (0.97, 1.01)	0.473
DM duration (>5 years)	1.36 (1.00, 1.83)	0.048	1.20 (0.84, 1.73)	0.313
Smoker/non‐smoker	1.05 (0.77, 1.42)	0.774	1.13 (0.72, 1.76)	0.596
Drinker/nondrinker	0.82 (0.60, 1.11)	0.194	0.75 (0.52, 1.1)	0.143
BMI 25–26/≤24	1.28 (0.91, 1.81)	0.159	1.16 (0.79, 1.71)	0.459
BMI ≥27/≤24	1.20 (0.84, 1.72)	0.311	0.93 (0.58, 1.49)	0.769
Waist circumference (>90/80 cm)	1.19 (0.90, 1.59)	0.230	1.18 (0.82, 1.72)	0.375
HbA_1c_ 7–9/HbA_1c_<7 (%), 53–75/<53 (mmol/mol)	1.16 (0.80, 1.70)	0.430	1.19 (0.8, 1.78)	0.393
HbA_1c_ ≥9/HbA_1c_<7 (%), ≥75/<53 (mmol/mol)	1.94 (1.34, 2.80)	0.001	1.92 (1.26, 2.93)	0.002
LDL>100 (mg/dl), >2.6 (mmol/L) or on drug treatment	1.02 (0.73, 1.43)	0.898	0.91 (0.65, 1.29)	0.610
TG >150/TG ≤150 (mg/dl), >1.7/ ≤150 (mmol/L)	1.21 (0.91, 1.60)	0.197	1.18 (0.87, 1.61)	0.279
BP≥140/90 or on drug treatment / BP<120/80 (mmHg)	1.65 (1.17, 2.31)	0.004	1.41 (0.90, 2.22)	0.137
Uric acid >7/≤7 (mg/dl), >416/≤416 (µmol/L)	1.09 (0.77, 1.52)	0.634	1.17 (0.80, 1.71)	0.418
HOMA‐IR				
Quartile 2 (1.5–3.4)/Quartile 1 (< 1.5)	1.40 (0.94, 2.07)	0.095	1.35 (0.90, 2.04)	0.153
Quartile 3 (3.5–4.9)/Quartile 1 (< 1.5)	1.64 (1.11, 2.41)	0.013	1.57 (1.04, 2.36)	0.032
Quartile 4 (≥ 5)/Quartile 1 (< 1.5)	1.54 (1.03, 2.32)	0.037	1.45 (0.94, 2.23)	0.096
Anti‐diabetic use, *n* (%)				
Sulphonylurea (Yes vs. No)	1.22 (0.75, 1.98)	0.424	0.85 (0.5, 1.45)	0.557
Biguanide (Yes vs. No)	1.46 (0.99, 2.17)	0.059	1.28 (0.85, 1.93)	0.238
Thiazolidinedione (Yes vs. No)	1.09 (0.69, 1.71)	0.716	0.89 (0.54, 1.46)	0.634
a‐Glucosidase inhibitor (Yes vs. No)	1.43 (0.83, 2.47)	0.197	1.21 (0.67, 2.18)	0.527
Meglitinide (Yes vs. No)	1.22 (0.50, 2.96)	0.662	1.02 (0.4, 2.61)	0.960
Antihypertensive use, *n* (%)				
Angiotensin‐converting enzyme inhibitor/angiotensin II receptor blocker (Yes vs. No)	1.52 (1.15, 2.01)	0.004	1.32 (0.93, 1.87)	0.123
Calcium channel blocker (Yes vs. No)	1.17 (0.87, 1.57)	0.298	0.95 (0.67, 1.34)	0.762
β‐Blocker (Yes vs. No)	1.32 (0.99, 1.76)	0.063	1.22 (0.86, 1.71)	0.263
Diuretic (Yes vs. No)	1.31 (0.94, 1.82)	0.111	1.19 (0.82, 1.73)	0.373
Statin use (Yes vs. No)	0.52 (0.17, 1.62)	0.259	0.45 (0.14, 1.45)	0.182
hsCRP (mg/dl)	0.98 (0.74, 1.30)	0.903	0.85 (0.6, 1.21)	0.360
ECG abnormal (Yes vs. No)	1.23 (0.85, 1.76)	0.269	1.27 (0.86, 1.88)	0.228
eGFR <60/≥60 ml/min/1.73 m^2^	0.85 (0.42, 1.73)	0.663	1.01 (1.00, 1.02)	0.040
Heart rate				
70–74 vs. <70	1.76 (1.14, 2.71)	0.010	2.05 (1.32, 3.18)	0.001
75–80 vs. <70	1.91 (1.23, 2.96)	0.004	2.10 (1.32, 3.32)	0.002
>80 vs. <70	1.46 (0.93, 2.30)	0.101	1.62 (1.01, 2.59)	0.046

Table 4 shows the effects of resting heart rate on microalbuminuria according to Cox regression analysis among the people with and without hypertension. We found that, in comparison to those with an heart rate <70 beats/min, only people with hypertension had a significantly increased microalbuminuria risk across those with heart rates of 70–74, 75–80 and >80 beats/min, with the associated hazard ratios being 2.38 (95% CI 1.36, 4.17), 2.33 (95% CI 1.29, 4.20) and 2.02 (95% CI 1.12, 3.64) respectively. A similar result was also found for the continuous heart rate data (hazard ratio=1.24, 95% CI:1.05, 1.47, indicating an average increased risk of microalbuminuria for increment of 10 beats/min was about 24% among those with hypertension).

**Table 4:** Effects of heart rate on microalbuminuria development according to Cox regression analysis among patients with and without hypertension

	With hypertension				Without hypertension			
	Univariate hazard ratio	*p*	Multivariable hazard ratio	*p*	Univariate hazard ratio	*p*	Multivariable hazard ratio	*p*
Heart rate								
70–74 vs. <70	1.95 (1.14, 3.34)	0.015	2.38 (1.36, 4.17)	0.003	1.79 (0.86, 3.70)	0.120	2.20 (0.99, 4.91)	0.053
75–80 vs. <70	1.94 (1.12, 3.38)	0.019	2.33 (1.29, 4.20)	0.005	2.09 (1.00, 4.37)	0.049	2.32 (1.02, 5.24)	0.044
>80 vs. <70	1.64 (0.95, 2.84)	0.076	2.02 (1.12, 3.64)	0.019	1.23 (0.55, 2.76)	0.621	1.29 (0.52, 3.21)	0.587
Heart rate (per 10 beats/min) (continuous)	1.19 (1.01, 1.39)	0.036	1.24 (1.05, 1.47)	0.014	1.00 (0.78, 1.29)	0.980	1.02 (0.74, 1.32)	0.911

**Figure 2:**
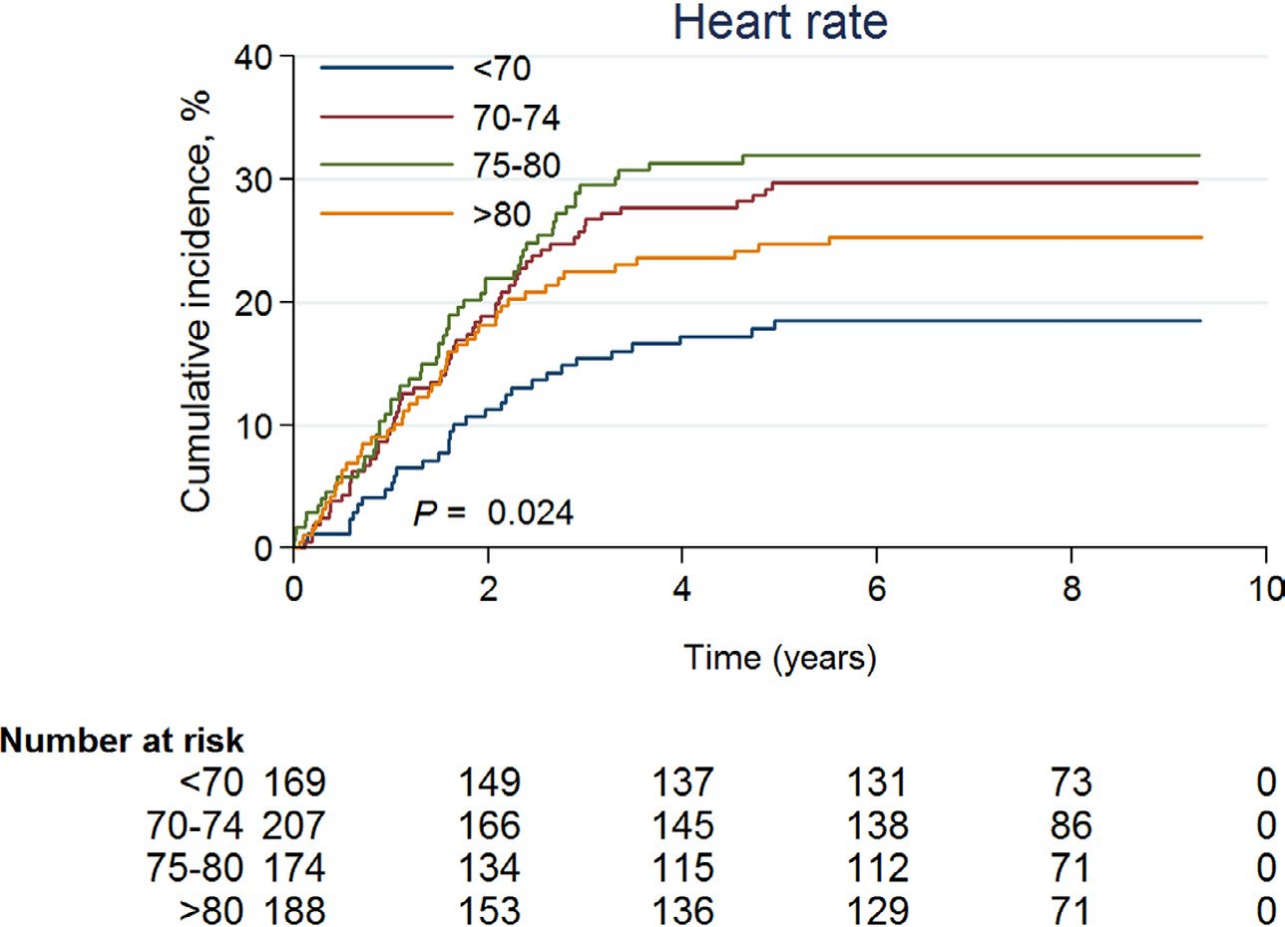
Cox proportional hazards regression analysis results for the cumulative incidence of microalbuminuria in patients with different heart rate ranges, with adjustments made for age, sex, age at diabetes diagnosis, education, smoking status, drinking status, BMI, waist circumference, HbA_1c_ level, LDL cholesterol level, triglyceride level, systolic blood pressure, diastolic blood pressure, uric acid level, HOMA‐IR, anti‐diabetic drug use, anti‐hypertension drug use, statin use, hsCRP level, eGFR level and EKG abnormal status

The cumulative incidence trends for the development of microalbuminuria in people with different heart rates are shown in Figure 2. To explore the robustness of our results, we further conducted two additional analyses to determine whether a higher resting heart rate is also a risk of development of macroalbuminuria and deterioration of renal function. Table S1 shows the adjusted hazard ratio of macroalbuminuria for those with resting heart rate of 70–74, 75–80 and >80 beats/min was 1.72 (0.98, 3.03), 1.09 (0.58, 2.05) and 1.99 (1.12, 3.51) respectively; the corresponding adjusted hazard ratio of eGFR < 60 ml/min/1.72 m^2^ was 1.51 (1.01, 2.27), 1.70 (1.13, 2.55) and 1.62 (1.09, 2.41) respectively.

## 4 DISCUSSION

Because the onset of CKD is insidious, and because it progresses slowly, the disease can easily escape notice. However, one sensitive indicator of early renal function injury is the urine ACR. An individual's resting heart rate, meanwhile, is a reliable indicator of his or her sympathetic nervous system excitation, and epidemiological research has shown that increases in resting heart rate are closely linked to the risks of diabetes and hypertension.^20^ Relatedly, the results of the present study showed a clear association between resting heart rates and urine ACRs of the type 2 diabetes people enrolled in the DMIDS project. More specifically, the results showed a positive correlation between resting heart rates and urine ACRs. Compared to the people in the first quartile (<70 beats/min), the risks of having a microalbuminuria were significantly increased for people in the second (70–74 beats/min), third (75–80 beats/min), and fourth (>80 beats/min) quartiles, being increased by 105% (hazard ratio = 2.05, 95% CI:1.32, 3.18), 110% (hazard ratio = 2.10, 95% CI:1.32, 3.32) and 62% (hazard ratio = 1.62, 95% CI:1.01, 2.59), respectively, even after adjusting for confounding factors.

According to a previous study conducted in Germany that included 4447 people with type 2 diabetes and at least one cardiovascular risk, as the heart rate increases, the risk of microalbuminuria is also increased,^21^ and the results of our study were consistent with this earlier finding. Besides, after controlling for more confounding factors, the current study reconfirmed that this risk association could also be applied to those without any additional cardiovascular risk. Meanwhile, another study extended those of the German study to a population of people with cardiovascular disease by showing that heart rate is associated with renal disease risk, thereby likewise suggesting that reducing the heart rate of such people might protect their kidney function.^2^ In this study, in contrast, we included a population with type 2 diabetes but free of obvious cardiovascular diseases, stratifying these people in terms of their blood pressure. The results showed that among people with hypertension, those in the second resting heart rate quartile (70–74 beats/min), third quartile (75–80 beats/min) and fourth quartile (>80 beats/min) tended to have higher urine ACRs, with the associated hazard ratios being 2.38 (95% CI: 1.36, 4.17), 2.33 (95% CI: 1.29, 4.20) and 2.02 (95% CI: 1.12, 3.64) respectively. Among those without hypertension, only those in the second resting heart rate quartile seemed to have higher urine ACRs, with a hazard ratio of 2.32 (95% CI: 1.02, 5.24). These interaction results suggest that the effects of hypertension itself on urine ACR may be responsible for the observed association between resting heart rate and urine ACR.

Our study results further showed that hypertension (hazard ratio: 1.66) is more significantly related to urine ACR than is resting heart rate (the hazard ratio for the highest resting heart rate quartile compared to the lowest quartile was 1.17). There is a positive relationship between resting heart rate and urine ACR in the general population, whereas this relationship disappears in people with both hypertension and diabetes.^22^ Combining this finding with the results of previous studies, we hypothesize that the relationship between resting heart rate and urine ACR may be restored in such people after providing them with effective hypoglycaemic and antihypertensive therapy, but further clinical trials would be required to confirm that speculation.

In any case, it seems clear that resting heart rate may serve as a marker of urine ACR development, which in turn is an indicator of early renal impairment. A number of mechanisms may underlie these effects; for example, the progression of atherosclerosis, and, in turn, nephrosclerosis, can occur as a result of alterations in endothelial oxidative stress, which are themselves sensitive to reductions in resting heart rate.^14^ There is also an association between resting heart rate and increased aortic stiffness, which in turn has associations with a number of cardiovascular disease outcomes, including heart failure and stroke,^23^ while an elevated resting heart rate has been shown to be correlated with both complications of renal failure and microvascular disease in general.^24^, ^25^ A number of previous studies have also demonstrated that, in addition to causing endothelial cell injury due to increased tensile stress, an elevated resting heart rate also increases the degree to which endothelial cells are permeable to circulating inflammatory mediators, thereby mediating the progression of microalbuminuria.^26^

In the kidney itself, studies have shown that myogenic autoregulation, which can serve to prevent hyperperfusion, is impaired in individuals with old age, hypertension and diabetes, making the kidneys of such individuals more prone to experiencing pulsatile stress.^27^, ^28^ At the same time, direct experimental research clarifying the specific effects and relevance of resting heart rate in humans have thus far been lacking. However, a previous study indicated that an elevated resting heart rate may be a biomarker of increased sympathetic activation,^29^ and greater sympathetic activation has previously been reported to occur in people experiencing renal failure.^30^ The kidney is not only a producer of sympathetic activity; rather, it also acts as a receiver of efferent signals. Increased sympathetic activity of the kidneys results in a cascade of subsequent responses: first, it causes angiotensin II to be produced by spurring the release of renin from juxtaglomerular cells. Angiotensin II, in turn, results directly in renal vasoconstriction, which in turn causes the renal blood flow and glomerular filtration rates to be decreased. These outcomes are further enhanced by direct activation of the renin—angiotensin system caused by kidney injury. Meanwhile, renal tubular sodium reabsorption is also directly enhanced as a result of any increases in renal sympathetic nerve activity.^31^

The participants in our study consisted of people in Taiwan with type 2 diabetes, suggesting that the study results should be only representative of such people. Compared to the participants in other studies, the participants in the present study also had relatively diverse blood pressure and blood glucose levels; therefore, we fully adjusted for most clinical relevant confounding variables as listed in Table 1 to make our results as robust as possible.

## 5 CONCLUSIONS

This study reported the association between resting heart rate and new‐onset microalbuminuria in people with type 2 diabetes, particularly for those with hypertension. After adjusting for blood pressure, blood glucose level, insulin resistance, inflammatory biomarkers and other confounding factors, our study persistently indicates higher resting heart rate could be a risk for people with type 2 diabetes in developing microalbuminuria, macroalbuminuria, and renal function decline. In clinical settings, physicians should pay more attention in identifying this high‐risk population for further preventive measures.

## COMPETING INTERESTS

The authors declare no potential conflicts of interest with respect to the research, authorship and/or publication of this article.

## AUTHOR CONTRIBUTIONS

Y.K.C. and C.C.H. designed the study and drafted the manuscript. Y.K.C. interpreted the data and statistical analyses. Y.K.C. and H.C.F. interpreted the results. Y.K.C., H.C.F. and C.C.H. revised and finalized the manuscript. All the authors approved the final manuscript.
