# Supplementary material for: The relationship between resting heart rate and new‐onset microalbuminuria in people with type 2 diabetes: An 8‐year follow‐up study
Source: Diabet Med. 2020 Nov 18;38(5):e14436. doi: 10.1111/dme.14436 (PMC8246976; doi:10.1111/dme.14436)
Supplement: Supplementary file 1 [file DME-38-0-s001.docx]

Supplemental Table. Univariate and adjusted Cox proportional hazards model to assess risk of macroalbuminuria and eGFR decline according to quartiles of baseline resting heart rate

|  | **Univariate**  **hazard ratio** | ***p*** | **Multivariable**  **hazard ratio*** | ***p*** |
| --- | --- | --- | --- | --- |
| **Risk of macroalbuminuria (urine ACR≥300 mg/g)†** |  |  |  |  |
| Resting heart rate (beats/min) |  |  |  |  |
| 70-74 vs. <70 | 1.66(0.96, 2.90) | 0.072 | 1.72(0.98, 3.03) | 0.060 |
| 75-80 vs. <70 | 1.17(0.63, 2.15) | 0.617 | 1.09(0.58, 2.05) | 0.784 |
| >80 vs. <70 | 1.93(1.12, 3.35) | 0.019 | 1.99(1.12, 3.51) | 0.018 |
|  |  |  |  |  |
| **Risk of eGFR decline (< 60 ml/min/1.73 m^2^)‡** |  |  |  |  |
| Resting heart rate (beats/min) |  |  |  |  |
| 70-74 vs. <70 | 1.30(0.88, 1.94) | 0.188 | 1.51(1.01, 2.27) | 0.043 |
| 75-80 vs. <70 | 1.47(0.99, 2.19) | 0.057 | 1.70(1.13, 2.55) | 0.010 |
| >80 vs. <70 | 1.66(1.13, 2.44) | 0.010 | 1.62(1.09, 2.41) | 0.017 |

* The multivariable models were adjusted for all covariates listed in Table 1.

† We selected those who had baseline urine ACR<300 mg/g (n=1094) to evaluate the effect of resting heart rate on macroalbuminuria development.

‡ We selected those who had baseline eGFR> 60 ml/min/1.73 m^2^ (n=1103) to evaluate the effect of resting heart rate on eGFR decline.
